# Increasing lysergic acid levels for ergot alkaloid biosynthesis: Directing catalysis *via* the F-G loop of Clavine oxidases

**DOI:** 10.3389/fmicb.2023.1150937

**Published:** 2023-03-16

**Authors:** Li Rong Lim, Garrett Wong, Maybelle K. Go, Wen Shan Yew

**Affiliations:** ^1^Synthetic Biology for Clinical and Technological Innovation, National University of Singapore, Singapore, Singapore; ^2^Synthetic Biology Translational Research Programme, Yong Loo Lin School of Medicine, National University of Singapore, Singapore, Singapore; ^3^NUS Graduate School—Integrative Sciences and Engineering Programme (ISEP), National University of Singapore, Singapore, Singapore; ^4^Department of Biochemistry, Yong Loo Lin School of Medicine, National University of Singapore, Singapore, Singapore

**Keywords:** ergot alkaloids, Clavine oxidases, cytochrome P450, elymoclavine, lysergic acid

## Abstract

Most ergot alkaloid drugs are semi-synthetically derived from the natural product lysergic acid, a valuable precursor for the development of novel ergot alkaloid drugs. Clavine oxidase (CloA) is a putative cytochrome P450, identified in the ergot alkaloid biosynthesis pathway, and a key enzyme that catalyzes the formation of lysergic acid from the precursor alkaloid agroclavine in a two-step oxidation reaction. We demonstrated in this study that *Saccharomyces cerevisiae* can be used as a viable host for the functional expression of CloA from *Claviceps purpurea* and its orthologs. We also showed that CloA orthologs differ in their ability to oxidize the substrate agroclavine, with some orthologs only able to perform the first oxidation reaction to produce elymoclavine. Of particular note, we identified a region between the F-G helices of the enzyme that may be involved in directing oxidation of agroclavine by substrate recognition and uptake. Using this knowledge, engineered CloAs were shown to produce lysergic acid at levels exceeding that of wildtype CloA orthologs; a CloA variant, chimeric AT5 9Hypo CloA, increased production levels of lysergic acid to 15 times higher as compared to the wildtype enzyme, demonstrating future utility for the industrial production of ergot alkaloids using biosynthetic routes.

## 1. Introduction

Alkaloids are a diverse group of nitrogen-containing compounds that are produced as secondary metabolites in plants and fungi. A subclass of alkaloids known as the ergot alkaloids are produced in several species of filamentous fungi in the phylum Ascomycota. The most significant member of this group is the parasitic fungus *Claviceps purpurea* of the genus *Claviceps*. Ergot alkaloids share structural similarities with neurotransmitters such as dopamine, noradrenaline, and serotonin, and it is not surprising that ergot alkaloids can act either agonistically or antagonistically on the same receptors as these neurotransmitters. All ergot alkaloids share a common tetracyclic ring system called the ergoline ring with the differences mainly in the variable R-group. The variable R-group allows the characterization of ergot alkaloids into three main groups of increasing structural complexity: (1) clavine alkaloids, (2) simple lysergic acid amide derivatives, and (3) ergopeptines (Schiff, 2006).

Of interests are the clavine alkaloids elymoclavine, lysergol, and lysergic acid, which are used as the starting materials for the semi-synthetic derivatization of ergot alkaloid drugs such as nicergoline and pergolide (Liu and Jia, 2017). Nicergoline is used for the treatment of cognitive impairment in various forms of dementia (Winblad et al., 2008), while pergolide is used as a first-line treatment to reduce tumor sizes in prolactinomas (Capozzi et al., 2015).

It was established in strains of *C. purpurea* that agroclavine was the precursor to elymoclavine and elymoclavine was the precursor to lysergic acid (Agurell and Ramstad, 1962). Studies using microsomal preparations of the strain *C. purpurea* PRL 1980 revealed that the reaction of agroclavine to elymoclavine was oxygen and NADPH dependent, and it was suggested that a mixed function oxygenase was involved in the oxidation reaction (Hsu and Anderson, 1970). It was also shown in the microsomal fraction of *C. purpurea* strain PRL 1980 that conversion of agroclavine to elymoclavine was inhibited by carbon monoxide and showed that the photochemical action spectrum had a corresponding maximum at 450 nm, a characteristic of P450 enzymes (Kim et al., 1981).

A gene encoding a putative cytochrome P450 in the ergot alkaloid synthesis (EAS) gene cluster of *C. purpurea* strain P1 was identified and was later designated *cloA* (Clavine Oxidase A; Haarmann et al., 2005). Disruption of the gene encoding *cloA* in *C. purpurea* strain P1 resulted in a strain that accumulated agroclavine and elymoclavine (Haarmann et al., 2006). This observation also led the authors to suggest that CloA was an elymoclavine oxygenase and that agroclavine oxygenase lies not in the EAS gene cluster but elsewhere in the *Claviceps* genome. In a more recent study, a heterologous fungal expression system was designed using an *Aspergillus fumigatus* strain expressing the *Epichloë* sp. Lp1 *easA-cloA* combination. This strain was able to produce lysergic acid from agroclavine, suggesting that the *Epichloë* sp. Lp1 CloA can perform two successive oxidation steps and an isomerization reaction prior to the release of the lysergic acid (Robinson and Panaccione, 2014). It remains unclear why some CloA orthologs were able to perform a one-step oxidation from elymoclavine to lysergic acid and the ability of others to perform a two-step oxidation reaction from agroclavine to lysergic acid.

In the present study, we used a sequence similarity network (SSN) approach to identify CloA orthologs from various species of the *Clavicipitaceae* family and showed that cloA orthologs can be functionally expressed in *S. cerevisiae*, underscoring the importance of the organism as a viable expression host. We also demonstrated that these orthologs exhibited different oxidation profiles when incubated with agroclavine and identified a region of the enzyme that plays a role in directing the catalytic activity of CloA. Utilizing this knowledge, we engineered variants of CloA with enhanced catalysis and increased the production levels of lysergic acid as compared to wildtype enzyme.

## 2. Materials and methods

### 2.1. Selecting cloA orthologs using a sequence similarity network

A sequence similarity network (SSN) was generated using the EFI-EST tool (Gerlt et al., 2015) using the protein sequence from *C. purpurea* cloA (Cpur) as the input and the number of retrieved sequences was limited to 5,000 sequences. An alignment score of 160 corresponding to 50% sequence identity was chosen for the generation of the SSN, and the SSN was visualized using Cytoscape, a software platform for visualizing complex networks (Shannon et al., 2003). Nodes were filtered by using text filters “Clavine” and “cloA” and “Claviceps,” followed by expanding the network to include the node’s first neighbors. The network was further refined, by removing uncharacterized nodes and nodes sharing identical amino acid sequences. A total of 15 nodes were selected for gene synthesis.

### 2.2. Synthesis and cloning of CloA orthologs

Selected orthologs of wild-type CloA were codon optimized for expression in *S. cerevisiae*, synthesized (GenScript, United States), and cloned into a pYES2/CT yeast expression vector (Invitrogen, United States) between the *BamHI* and *XhoI* restriction sites. Chimeric mutants of cloA and alanine scanning mutants were PCR amplified using primers listed in Supplementary Table S1 and assembled into pYES2/CT yeast expression vectors, between the *BamHI* and *XhoI* restriction sites using Gibson assembly (New England Biolabs, United States) following the manufacturer’s directions. Cloned expression vectors harboring the chimeric cloAs were sequenced using GAL1 Promoter and CYC1 Terminator primers.

### 2.3. Transformation, expression, and screening of cloA in *Saccharomyces cerevisiae* strain BJ2168

Expression vectors with the *cloA* genes were transformed into the strain BJ2168 (ATCC® 208,277) using a modified method that was previously described (Gietz and Schiestl, 2007). Individual colonies of transformed cells were inoculated into 4.5 ml of Synthetic Complete-Uracil (SC-Ura) 0.1% (w/v) glucose and incubated at 30°C for 30 h. After 30 h, yeast cells were induced with 500 μL of 20% (w/v) galactose supplemented with 10 mM FeCl_3_ and 10 mM 5-aminolevulinic acid (5-ALA) to a final concentration of 2% galactose (w/v), 1 mM FeCl_3_ and 1 mM 5-ALA. Cells were incubated at 25°C for another 24 h for protein expression. Post-induction, 998 μL of induced cells were transferred to a 96 deep well block and 2 μL of 2.1 mM agroclavine (Chiron AS, Norway) was added to a final concentration of 4.2 μM. Cells were incubated at 25°C for another 24 h. Cell suspension was spun down at 4000 rpm for 10 min and 200 μL of the supernatant were filtered through a 96 well MultiScreen® Solvinert filter plate.

### 2.4. Liquid chromatography and mass spectrometry

Samples were separated using an Agilent InfinityLab Poroshell 120 EC-C18 column with the dimensions of 2.1 mm × 100 mm, 1.9 μm particle size coupled to an Agilent 1290 Infinity liquid chromatography system. All solvents used were LC-MS grade, mobile phases used consisted of (A) water with 0.1% (v/v) formic acid and (B) acetonitrile with 0.1% (v/v) formic acid at a flow rate of 0.2 ml∙min^−1^. Sample injection volume was 5 μL, and separation was carried out using a linear gradient starting from a pre-equilibrated solvent ratio of 95:5 (A:B) to a solvent ratio of 5:95 (A:B) over 6 min, the column was washed with a solvent ratio of 5:95 (A:B) over 2 min before equilibrating to a solvent ratio of 95:5 (A:B) for another 1 min.

Mass spectrometry was carried out using an Agilent Technologies 6550 iFunnel Q-TOF with a Dual AJS electrospray ionization source with the following conditions: gas temperature of 200°C, drying gas 15 L∙min^−1^, nebulizing gas pressure of 50 psi, sheath gas temperature of 350°C, a sheath gas flow of 11 L∙min^−1^, capillary voltage of 2,500 V, and a nozzle voltage of 2,000 V.

Targeted MS/MS was performed using fixed polarity (positive), from the eluent beginning from 1 min into the run. Instrument parameters were set to run at; source gas temperature and flow of 200°C and 10 L min^−1^, sheath gas temperature and flow of 350°C and 10 L min^−1^, nebulizer pressure at 50 psig. Capillary and nozzle voltages were set to 4,000 V and 0 V, respectively. MS1 was set to a mass range of 40–1,000 m/z, at a scan rate of 3 spectra/s. MS2 was set to a mass range of 40–1,000 m/z, at a scan rate of 6 spectra s^−1^ using a fixed collision energy of 20 *e*V. The targeted masses were set for: (1) 239.1543 m/z, narrow isolation width (1.3 amu); (2) 269.1285 m/z, narrow isolation width (1.3 amu); and (3) 255.1492 m/z, narrow isolation width (1.3 amu).

Determination of compound identities was performed by the comparison of retention time and MS/MS product ion spectrum against commercially obtainable standards where available (*D*-lysergic acid; Chiron; agroclavine; Chiron/Toronto Research Chemicals; elymoclavine; Toronto Research Chemicals; lysergol; Toronto Research Chemicals).

## 3. Results and discussion

### 3.1. Sequence similarity network of CloA

**Figure 1:**
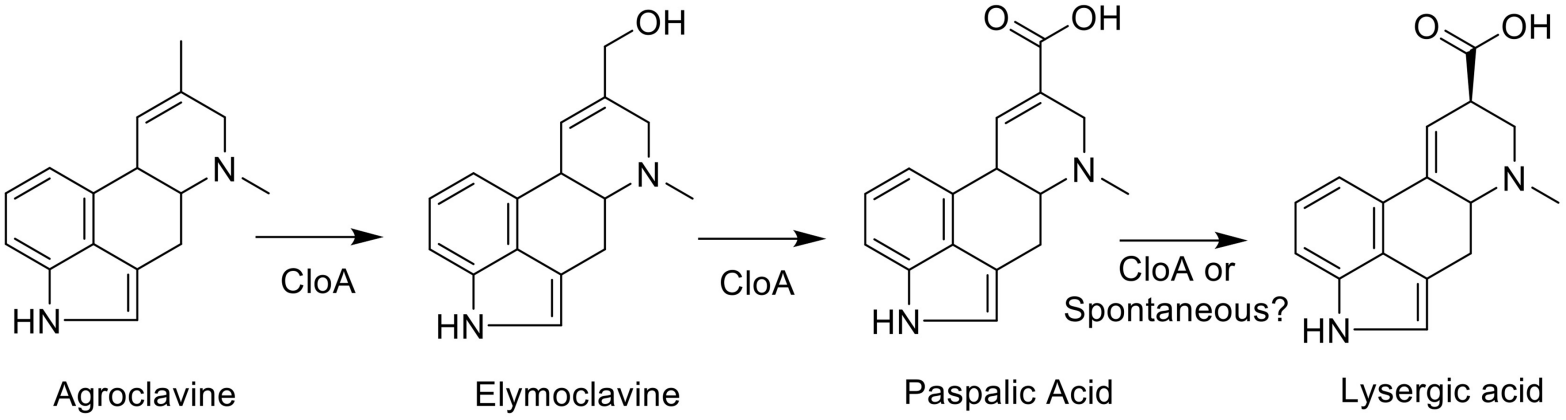
Clavine oxidase (CloA) catalyzes multiple oxidation steps of agroclavine to form elymoclavine and thereafter lysergic acid.

To probe the mechanism of the two-step oxidation reaction of CloA (Figure 1), an SSN was generated using the EFI-EST sequence BLAST function, using the protein sequence from Cpur CloA as the input and the number of retrieved sequences was limited to 5,000 sequences. In an SSN, protein sequences that are highly similar share a high degree of interconnectivity and form clusters. A user can infer the function of an uncharacterized enzyme by associating it with enzymes of known function in the same cluster (Gerlt et al., 2015).

**Figure 2:**
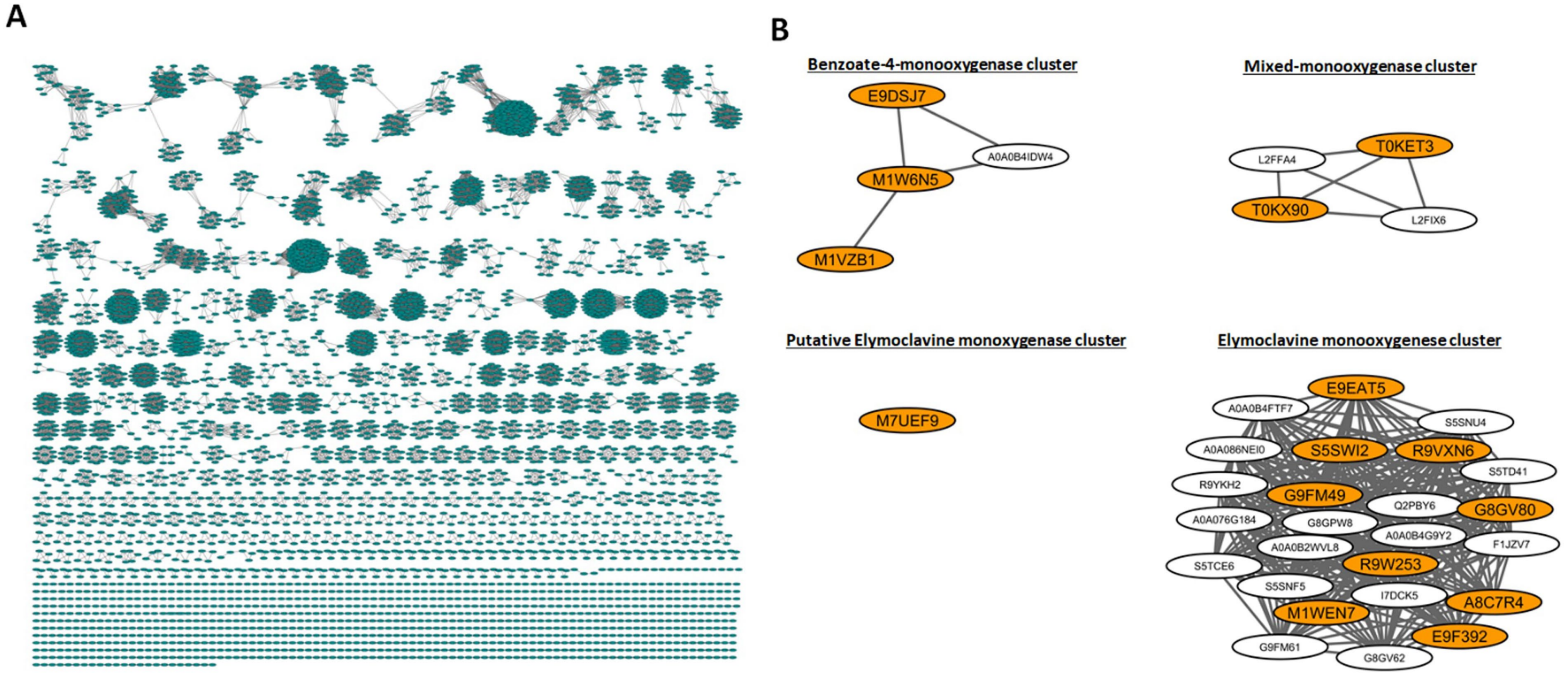
Sequence similarity network (SSN) and selection of cloA genes. **(A)** Full SSN network generated from EFI-EST. **(B)** Node clusters containing nodes with the descriptors “Claviceps” “Clavine” or “cloA,” selected nodes are highlighted in orange.

The full SSN of CloA comprised 4,998 nodes and had 37,424 edges; text filters “Clavine” and “Claviceps” were used to select nodes that contained these descriptions. We expanded the selection to include the node’s first neighbors resulting in four clusters comprising of 77 nodes and 1,042 edges. Uncharacterized and redundant sequences were removed to refine the network to 34 nodes and 306 edges (Figure 2). Out of the 34 nodes, 15 genes with the following UniProt annotations were selected for study: M1WEN7 (Cpur), G8GV80 (Cpas), S5SWI2 (Nlo), E9F392 (392), E9EAT5 (AT5), A8C7R4 (C7R4), R9VXN6 (XN6), R9W253 (253), G9FM49 (9Hypo), T0KET3 (ET3), T0KX90 (X90), M7UEF9 (BOTF1), E9DSJ7 (SJ7), M1W6N5 (W6N5), and M1VZB1 (ZB1). A detailed description of the orthologs can be found in Supplementary Table S2.

### 3.2. Optimizing cofactor availability by addition of 5-aminolevulinic acid and FeCl_3_ improves lysergic production in *Saccharomyces cerevisiae*

A major concern for the overexpression of functional recombinant enzymes, such as cytochrome P450s, is the depletion of essential cofactors, such as heme and iron, which are essential components of the active site. To examine the effect of these cofactors on the quality of CloA expression, we performed a small-scale expression screening with Cpur CloA. We supplemented the medium with 5-aminolevulinic acid (5-ALA), the first intermediate in the biosynthesis of heme, and/or iron (III) chloride (FeCl3) during induction with galactose. Prior to screening the full complement of CloA orthologs, we wanted to determine if these supplements affected the activity of CloA.

**Figure 3:**
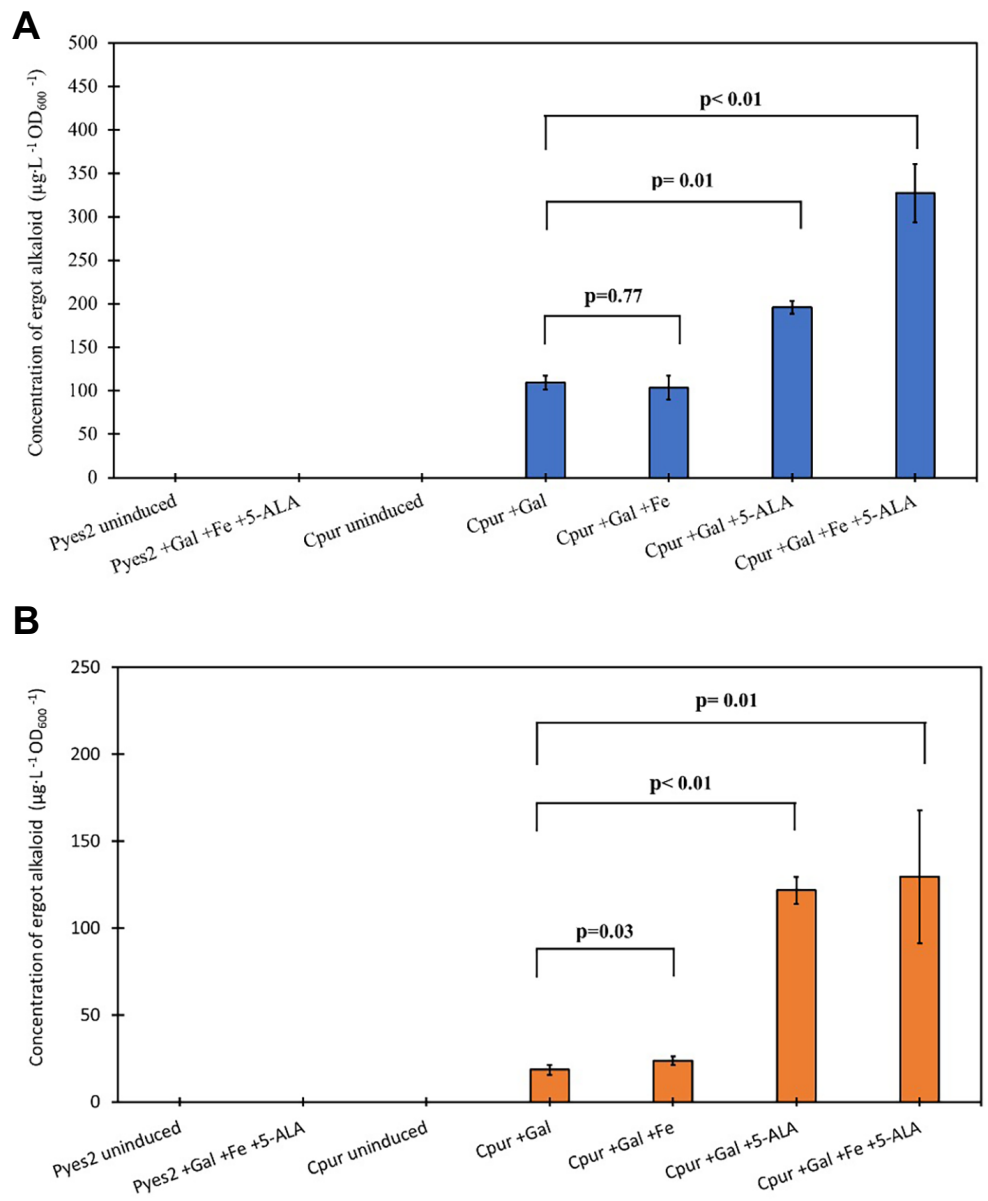
Screening the effects of supplement addition on CloA activity. Blue bars represent the mean concentration of lysergic acid while orange bars represent the mean concentration of elymoclavine produced by CloA orthologs. Error bars are representative of three biological replicates.

Supplementing the media with FeCl_3_ alone, did not improve production levels of Cpur CloA and showed comparable production levels of elymoclavine and lysergic acid with samples induced with galactose alone (Figure 3; Table 1). The addition of 5-ALA to the induction reaction mixture almost doubled the lysergic acid yield from 109.3 ± 7.8 μg∙L^−1^ OD_600_^−1^ in samples induced with galactose alone to 195.6 ± 7.3 μg∙L^−1^ OD_600_^−1^. The addition of 5-ALA also increased the yield of elymoclavine from 18.5 ± 2.8 μg∙L^−1^ OD_600_^−1^ in samples induced with galactose to 121.6 ± 7.7 μg∙L^−1^ OD_600_^−1^. A further increase in lysergic acid production was observed when both FeCl_3_ and 5-ALA were supplemented together into the induction mix to yield a concentration of 327.3 ± 33.5 μg∙L^−1^ OD_600_^−1^, almost three times more than induction with galactose alone.

**Table 1:** Summary of the effects of supplementation on the activity of Cpur cloA in the presence of agroclavine.

Expression condition	Mean concentration of lysergic acid (μg∙L^−1^ OD_600_^−1^) (±SD^α^)	Mean concentration of elymoclavine (μg∙L^−1^ OD_600_^−1^) (±SD^α^)
pYES2 uninduced	ND^*^	ND^*^
Cpur uninduced	ND^*^	ND^*^
Cpur + Gal	109.34 (± 7.80)	18.5 (± 2.83)
Cpur + Gal + Fe	103.42 (± 13.51)	23.74 (± 2.33)
Cpur + Gal + 5-Ala	195.57 (± 7.27)	121.61 (± 7.67)
Cpur + Gal + Fe + 5-Ala	327.31 (± 33.45)	129.47 (± 38.28)
pYES2 + Gal + Fe + 5-Ala	ND^*^	ND^*^

α SD, denotes standard deviation.

* ND, denotes no detectable activity.

The observed improvement of CloA activity upon the addition of both 5-ALA and FeCl_3_ during induction of expression suggested that both 5-ALA and FeCl_3_ are limiting during the expression of CloA in *S. cerevisiae*. Heterologous expression of CloA under the control of the strong inducible GAL1 promoter, can impose cellular stress by rapidly depleting intracellular heme levels, leading to increased protein misfolding and increased proteasomal degradation (Table 2).

**Table 2:** Summary of the activity of AT5 9Hypo cloA in the presence of agroclavine.

	Mean concentration of agroclavine (μM∙OD_600_^−1^) (±^α^SD)	Mean concentration of elymoclavine (μM OD_600_^−1^) (±^α^SD)	Mean concentration of lysergic acid (μM∙OD_600_^−1^) (±^α^SD)
pYES2	4.12 (± 0.263)	N.D^*^	N.D^*^
AT5	0.412 (± 0.185)	2.75 (± 0.098)	N.D^*^
9Hypo	N.D^*^	2.23 (± 0.173)	0.174 (± 0.014)
AT5 9Hypo	N.D^*^	0.113 (± 0.005)	2.62 (± 0.235)

α SD, denotes the standard deviation of three biological replicates.

* ND, denotes no detectable compound.

Heme depletion has been identified and attributed to the overexpression of P450 monooxygenases in *S. cerevisiae* (Michener et al., 2012). An increase in the amount of theophylline produced from caffeine when caffeine demethylase P450 was expressed with heme biosynthesis enzymes. This suggested that heme depletion was a limiting factor that affected the activity of heme containing enzymes when expressed in *S. cerevisiae*. Increased levels of proteasomal activity were observed in cultures expressing P450 from a high copy plasmid, and proteasomal activity decreased when the same P450 was expressed, with genes that increase heme biosynthesis (Michener et al., 2012). Taken together, this suggested that without the necessary components to form a holoenzyme (with the prosthetic group), the apoenzymes (without the prosthetic group) will be degraded, leading to a lower enzyme activity.

While the proteasomal activity of strains expressing CloA was not examined in this study, the expression of CloA under a strong promoter without supplementation could result in increased enzyme degradation. This could explain the lower production levels of Cpur CloA activity when induced with galactose alone, as well as the increased production levels in the presence of 5-ALA and FeCl_3_. Expression of CloA orthologs will be induced together with these supplements, for the rest of this study.

### 3.3. Chimeric AT5 9Hypo CloA was able to catalyze the second oxidation reaction

**Figure 4:**
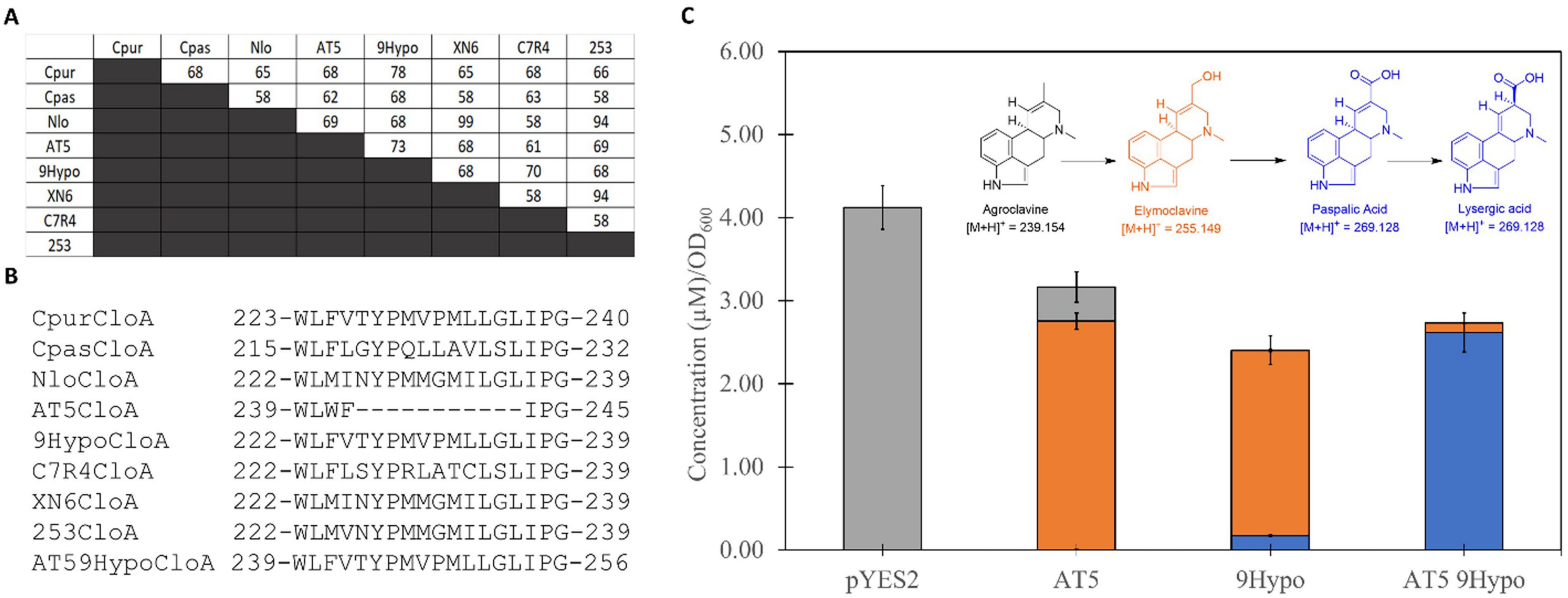
Sequence analysis of active CloA orthologs and sequence alignment. **(A)** Pairwise sequence alignment scores of active wildtype CloA sequences. **(B)** Multiple sequence alignment of active wildtype CloA sequences in the region of the missing sequence in AT5 cloA. Numbers represent the amino acid position in each wildtype orthologs. Sequence of the 9Hypo CloA orthologs was inserted into the region of AT5 CloA between the conserved residues WL and IPG. Numbers represent the amino acid position in each orthologs. **(C)** Screening of chimeric AT5 9Hypo CloA in *Saccharomyces cerevisiae* with agroclavine as a substrate. Error bars are representative of three biological replicates.

Applying the optimized expression conditions, we screened the 15 selected orthologs (Supplementary Figures S1, S2) and found that 8 of the 15 displayed activity on agroclavine. More interestingly, of the 8 active orthologs, 5 were able to produce lysergic acid and elymoclavine, while the remaining three were limited to producing elymoclavine. To gain insight into the differences in product profiles of the eight active CloA orthologs, we created a multiple sequence alignment. Pairwise sequence alignment of these sequences, which showed activity on agroclavine, revealed sequence identities ranging from 58% to 99% (Figure 4A). The highest pairwise sequence identity, between a single oxidation reaction ortholog and a double oxidation reaction ortholog, was between AT5 CloA and 9Hypo CloA. These two orthologs shared a 73% sequence identity. Notably, a gap of 11 amino acid residues was observed in the single oxidation producing orthologs AT5 CloA when its sequence was aligned to the other active CloAs (Figure 4B).

A chimeric AT5 CloA was constructed by inserting the sequence of 9Hypo CloA between the conserved residue pairs tryptophan-leucine (WL) and isoleucine-proline (IP). This chimeric AT5 CloA was termed AT5 9Hypo CloA and was screened for activity in the presence of the substrate agroclavine. The chimeric AT5 9Hypo CloA was now able to catalyze the second oxidation reaction (Figure 4C). The [M + H]^+^ mass of 255.15 m/z, which corresponded to the elymoclavine standard, and the [M + H]^+^ mass of 269.13 m/z, which corresponded to the lysergic acid standard, was observed in the extracted ion chromatograms of AT5 9Hypo CloA (Supplementary Figure S2).

The production levels of lysergic acid by this chimeric AT5 9Hypo CloA were measured to be 2.62 ± 0.235 μM∙OD_600_^−1^. This production level of lysergic acid was about 15 times higher compared to the wildtype 9Hypo CloA, which produced 0.174 ± 0.014 μM∙OD_600_^−1^ of lysergic acid (Figure 4C; Table 2).

**Table 3:** Summary of the activity of AT5 and AT5 9Hypo cloA Y134A mutants in the presence of agroclavine.

	Mean concentration of agroclavine (μM∙OD_600_^−1^) (±^α^SD)	Mean concentration of elymoclavine (μM∙OD_600_^−1^) (±^α^SD)	Mean concentration lysergic acid (μM∙OD_600_^−1^) (±^α^SD)
pYES2	3.76 (± 0.197)	N.D^*^	N.D^*^
AT5	0.399 (± 0.186)	3.49 (± 0.114)	N.D^*^
AT5 Y134A	2.49 (± 0.236)	N.D*	N.D^*^
AT5 9Hypo	N.D^*^	0.044 (± 0.014)	3.30 (± 0.338)
AT5 9Hypo Y134A	0.428 (± 0.055)	2.81 (± 0.207)	N.D^*^

α SD, denotes standard deviation of three biological replicates.

* N.D, denotes no detectable compound.

The production level of elymoclavine in AT5 9Hypo CloA was 0.113 ± 0.005 μM∙OD_600_^−1^. This production level was lower compared to wildtype AT5 CloA (2.75 ± 0.098 μM∙OD_600_^−1^) and wildtype 9Hypo CloA (0.174 ± 0.014 μM∙OD_600_^−1^). This decrease in elymoclavine production observed in the chimeric AT5 9Hypo CloA is likely attributed to the gain of function of the enzyme, as the chimeric enzyme was now able to utilize elymoclavine produced from the first oxidation reaction, as the substrate for the second oxidation reaction to produce lysergic acid.

### 3.4. Phyre2 modeling of AT5 CloA and AT5 9Hypo

There are no known CloA structures deposited into the PDB. A protein modeling approach was used as an attempt to understand why the inserted sequence in AT5 9Hypo CloA enabled the enzyme to catalyze the second oxidation reaction. The Phyre2 web server is a combination of several software suites that aims to provide the user with a friendly interface for protein modeling and bioinformatic analysis (Kelley et al., 2015). The Phyre2 webserver was used to generate a list of homology models of CloA that are based on existing structures in the PDB.

The top 10 templates of AT5 9Hypo CloA are summarized in Supplementary Table S3 and each template is accompanied by two important information, the confidence level, and the sequence identity, respectively. The confidence level (0% to 100%) represents the probability that the query is homologous to the template while the sequence identity is the proportion of the query residues that are identical to the template.

It was observed that the top 10 templates of AT5 9Hypo CloA share a very high confidence level (>90%), but very low sequence identity (<20%). This suggested that the core of the model or the relative positioning of the secondary structures are modeled with a high degree of confidence (RMSD 2 to 4 Å). However, the low sequence identity suggested that the models may show substantial deviations in the loops and non-core regions and any structure–function relationships derived from these templates would still require experimental validation.

Of the models generated by Phyre2, the models of AT5 9Hypo CloA that was based on the rat mitochondrial P450 24A1 (PDB ID: 3K9V) template stood out and showed (1) a clear substrate access channel and (2) a cleft on the surface of the enzyme bounded by the 11 inserted residues in AT5 9Hypo CloA (Supplementary Figure S5). These observations were not apparent in the other models of PDB ID: 1TQN and 4FDH, which each shared a 19% sequence identity and PDB ID: 3NA0 which shared an 18% sequence identity to AT5 9Hypo CloA. As such, the homology models of both wildtype and AT5 9Hypo CloA were based on PDB ID: 3K9V and chosen for downstream analysis and experimental validation.

### 3.5. Residues in the C-terminal end of the loop forms a cleft with Y134 that may be involved in elymoclavine recognition

**Figure 5:**
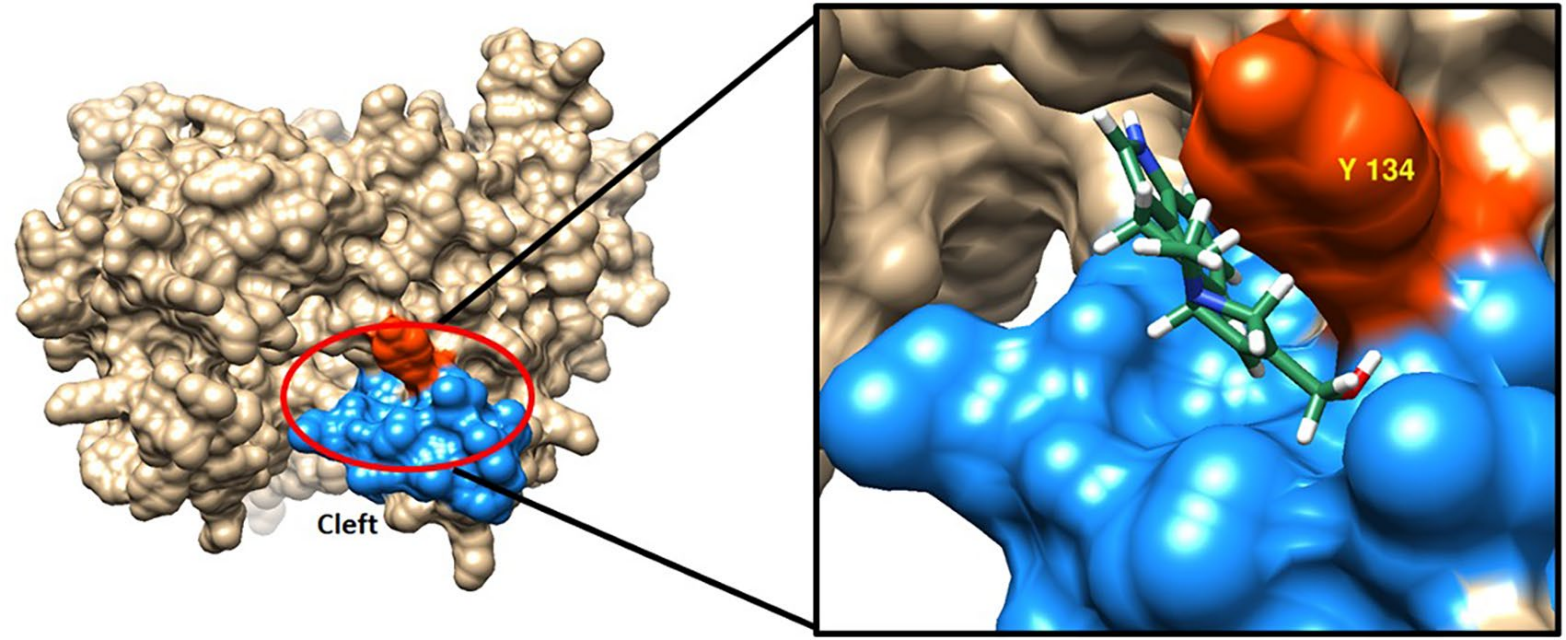
Surface representation of AT5 9Hypo CloA showing the residues in the F-G loop together with a protruding tyrosine residue potentially forming an elymoclavine binding site. The inserted residues are colored blue while the protruding tyrosine residue Y134 is highlighted in red (Left). Elymoclavine shown in ball and stick representation can be docked into this cleft (Insert).

In the Phyre2 model structures of AT5 9Hypo CloA, residues Y244 to P248 form a short loop, while residues M249 to L253 form a short G’ helix. Examination of the surface representation of the inserted region in both the models of AT5 CloA and AT5 9Hypo CloA, revealed a cleft on the surface of AT5 9Hypo CloA. This cleft was bounded by the residues in the F-G loop and a tyrosine residue Y134 that was orientated toward the exterior surface of the enzyme (Figure 5). The Autodock Vina function of Chimera was used to dock elymoclavine into this region and it was observed that elymoclavine fits into this cleft with a score of-7.3 kcal∙mol^−1^, suggesting a possible elymoclavine binding site at the surface of the enzyme (Figure 5-inset).

**Figure 6:**
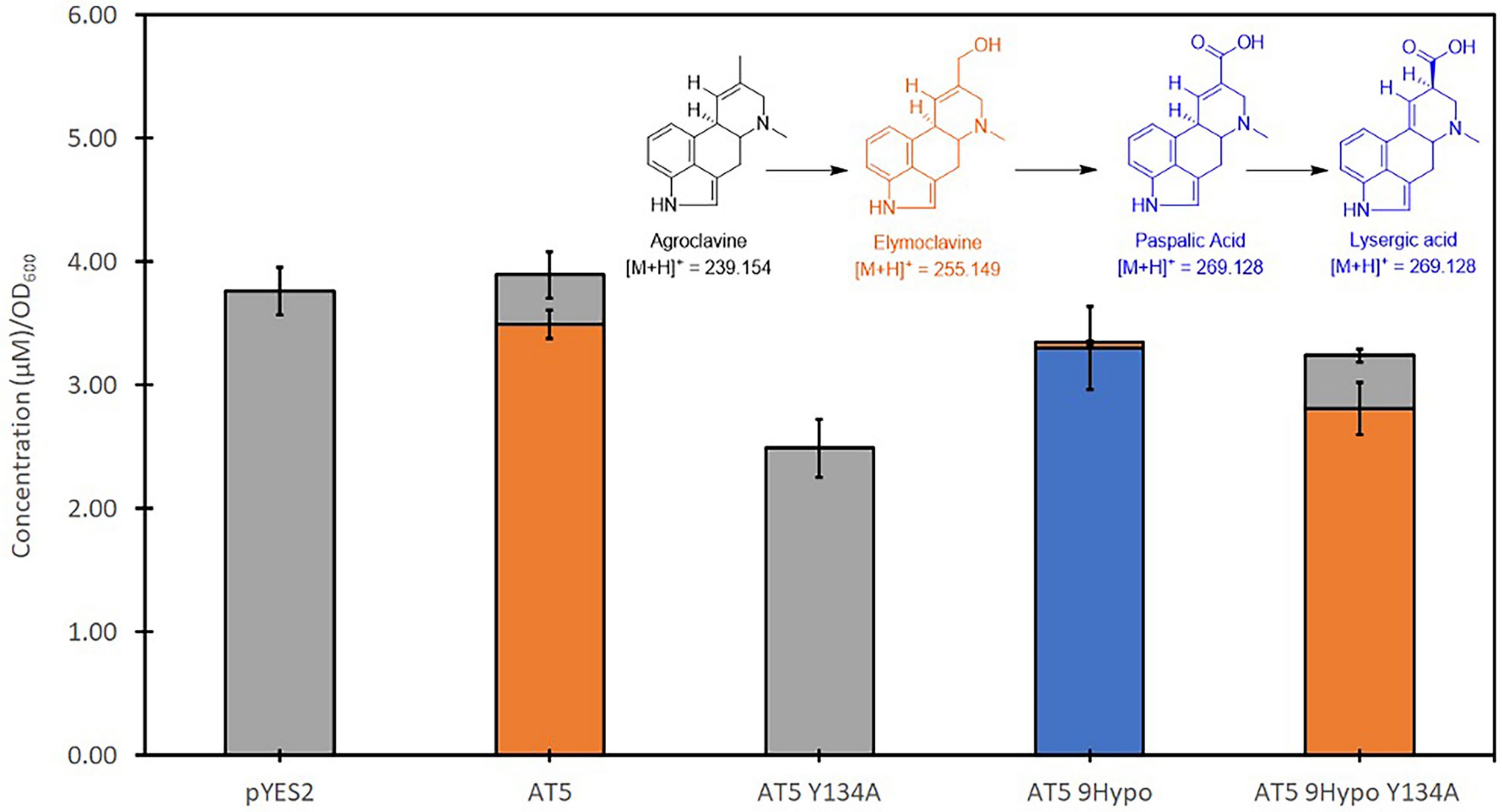
Screening of AT5 and AT5 9Hypo CloA Y134A mutants in the presence of agroclavine. Blue bars represent the mean concentration of lysergic acid while orange bars represent the mean concentration of elymoclavine produced by AT5 9Hypo CloA phenylalanine gate mutants. Error bars are representative of three biological replicates.

An alanine mutagenesis was performed on Y134 for both AT5 CloA and AT5 9Hypo CloA, respectively, to examine its role in the two-step oxidation of agroclavine. Interestingly, an alanine substitution in this residue in both AT5 CloA and AT5 9Hypo CloA resulted in a drastic change in the oxidation profiles. In AT5 CloA, the alanine mutant showed no turnover of agroclavine to elymoclavine, while the alanine mutant in AT5 9Hypo CloA showed a complete loss of lysergic acid production and only produced elymoclavine (Figure 6; Table 3).

**Table 4:** Summary of the activity of chimeric AT5 cloA in the presence of agroclavine.

	Mean concentration of agroclavine (μM∙OD_600_^−1^) (±^α^SD)	Mean concentration of elymoclavine (μM∙OD_600_^−1^) (±^α^SD)	Mean concentration lysergic acid (μM∙OD_600_^−1^) (±^α^SD)	% Elymoclavine of total detected alkaloids	% Lysergic acid of total detected alkaloids
pYES2	4.12 (± 0.263)	N.D^*^	N.D^*^	0.00	0.00
Cpur	N.D^*^	0.128 (± 0.024)	2.23 (± 0.350)	5.43	94.57
Cpas	N.D^*^	1.73 (± 0.231)	0.398 (± 0.069)	81.29	18.71
Nlo	N.D^*^	0.890 (± 0.219)	1.03 (± 0.164)	46.39	53.61
9Hypo	N.D^*^	2.23 (± 0.17)	0.174 (± 0.014)	92.77	7.19
XN6	N.D^*^	0.739 (± 0.15)	1.13 (± 0.115)	39.59	60.41
AT5 Cpur	N.D^*^	0.281 (± 0.036)	2.15 (± 0.118)	11.57	88.43
AT5 Cpas	N.D^*^	0.054 (± 0.003)	2.68 (± 0.143)	1.97	98.03
AT5 Nlo	N.D^*^	0.123 (± 0.037)	2.06 (± 0.185)	5.65	94.35
AT5 9Hypo	N.D^*^	0.113 (± 0.019)	2.62 (± 0.235)	4.14	95.86

α SD, denotes standard deviation of three biological replicates.

* ND, denotes no detectable activity in the presence of agroclavine.

The region between the F and G helices has been extensively observed in crystal structures and in molecular dynamics simulations to interact with substrates and the lipid bilayer membrane (Šrejber et al., 2018). In structural studies of camphor bound CYP101D2, the electron densities corresponding to multiple molecules of camphor were found in the camphor-soaked crystal structure. The electron density of one of these camphor molecules was found in a cavity on the F-helix side of the F-G loop, suggesting a multi-step binding mechanism whereby the substrate binds to a recognition site on the protein surface in the proximity of the F-G loop before entering the active site (Yang et al., 2010).

We hypothesize that Y134A in AT5 CloA could be essential for forming the early interactions with agroclavine at the surface of the enzyme, and loss of the Y134 side chain could result in a weaker interaction and a mutant that is unable to recognize or productively bind agroclavine within the active site of the enzyme. It is also hypothesized that Y134A in both AT5 CloA and AT5 9Hypo CloA may play a similar role in substrate recognition and binding. The difference, however, is that the extended F-G loop formed by the 11 inserted residues in AT5 9Hypo CloA may form interactions that is sufficient for the recognition of agroclavine but insufficient for the recognition of elymoclavine, which may help to explain the loss of the second oxidation reaction in this mutant. Further studies performed with the experimentally-determined structure of CloA would help to provide greater insight into this observation.

### 3.6. Substitution of residues from the other active wildtype CloAs

**Figure 7:**
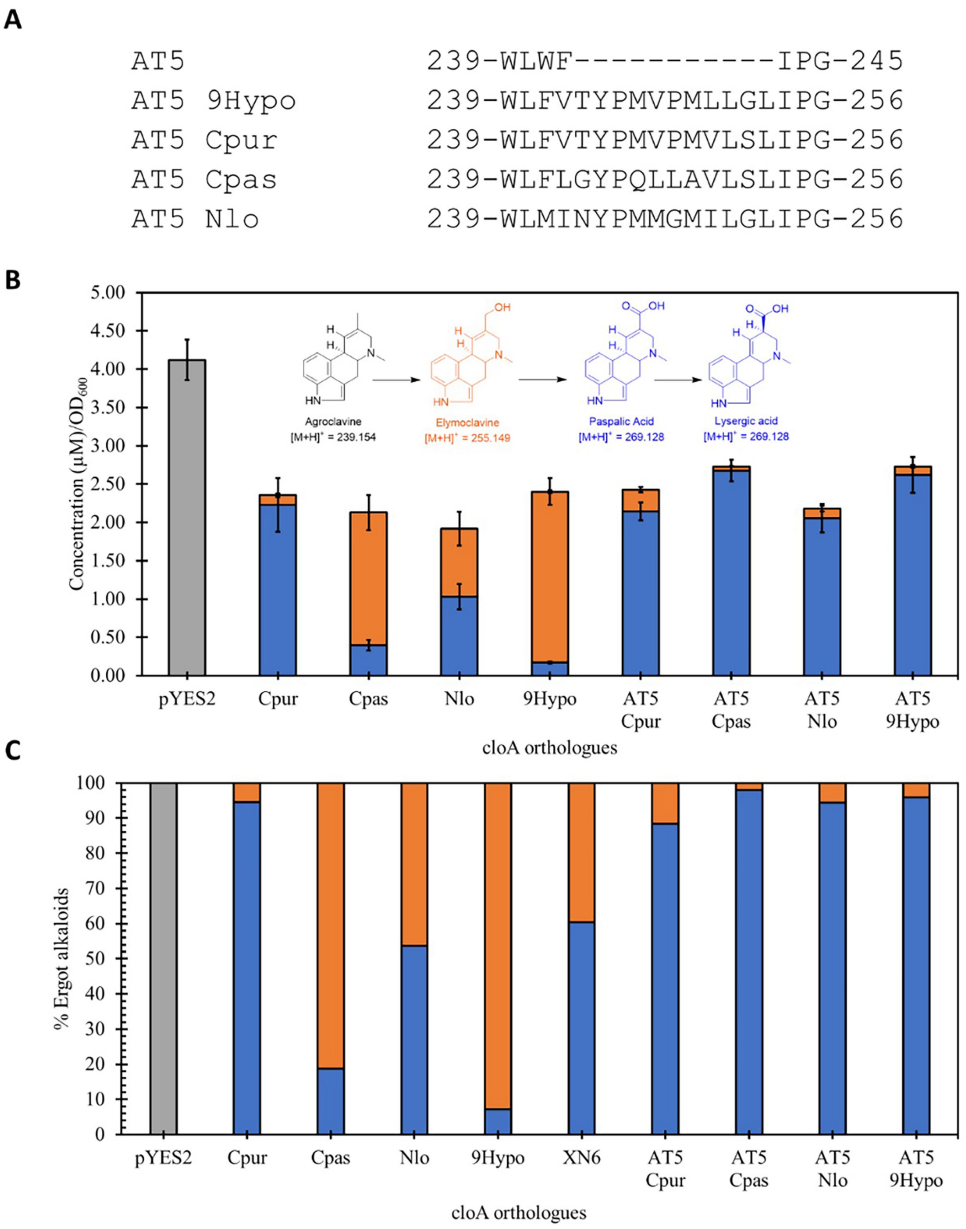
Screening of lysergic acid producing wildtype CloA and chimeric AT5 CloAs in the presence of agroclavine. **(A)** Sequence alignment of the inserted sequences from lysergic acid producing CloA into AT5 CloA. **(B)** Activity screening of chimeric AT5 CloAs. Blue bars represent the mean concentration of lysergic acid while orange bars represent the mean concentration of elymoclavine produced by CloA orthologs. Error bars are representative of three biological replicates. **(C)** Percentage of produced ergot alkaloids by lysergic acid producing wildtype CloA and chimeric AT5 CloAs.

To examine if the interaction is sequence specific, we further screened additional chimeric AT5 CloA mutants by inserting the 11 residues from the other CloA sequences onto the same AT5 CloA scaffold (Figure 7A). These new AT5 CloA chimeric mutants were tested for activity in the presence of agroclavine.

Interestingly, it was observed that these new AT5 CloA mutants were also able to perform a two-step oxidation reaction to produce lysergic acid (Figure 7B). The yields of each chimeric AT5 CloA mutant are summarized in Table 4. The best producers of lysergic acid between the chimeric AT5 CloAs were AT5 Cpas CloA (2.678 ± 0.143 μM∙OD_600_^−1^) and AT5 9Hypo CloA (2.62 ± 0.235 μM∙OD_600_^−1^). Compared to the best lysergic acid producer Cpur CloA (2.23 ± 0.350 μM∙OD_600_^−1^), these chimeric mutants exhibited an increase in the percentage of lysergic acid to the overall detected ergot alkaloids (Figure 7C): AT5 Cpas CloA produced 98.03% of lysergic acid and AT5 9Hypo CloA produced 95.86% of lysergic acid as compared to 94.57% of lysergic acid produced by Cpur CoA. While the reasons for this observation are unclear, one possible explanation is that the residues of Cpas and 9Hypo CloA could form a surface topology that forms a more efficient interaction with elymoclavine at the enzyme surface. Solving the crystal structures of both wildtype and chimeric CloAs with substrates or analogs that fully occupy the enzyme’s putative binding sites would provide important insights to better explore this hypothesis.

A second possible explanation is the interaction between the residues in this region and the membrane. Eukaryotic cytochrome P450s are usually membrane-bound enzymes that form complex relationships with both the membrane, and other membrane-bound redox partners (Šrejber et al., 2018). Using a combination of molecular dynamics simulations supported by experimental data, the F-G loop of CYP101D2 has been identified as one region that interacts with the membrane (Baylon et al., 2013). This interaction helps to modulate the depth of membrane insertion and the uptake of substrates from the membrane.

To further examine the effect of loop deletions on catalysis, loop deletion mutants (DeLoop) in wildtype CloA orthologs were created and assayed for activity (Supplementary Figure S6; Supplementary Table S4). It was observed that all the loop deletion mutants of wildtype CloA orthologs were unable to catalyze the oxidation of agroclavine. A corollary expectation is that modifications of the F-G loop region in CloA could affect its association with the membrane and modulate access of the substrate into the active site of the CloA.

## 4. Summary

Clavine oxidases (CloA) are a group of putative cytochrome P450s that are involved in the multiple step oxidations of agroclavine in the ergot alkaloid pathway. In this study, we combined the use of SSN, heterologous protein expression, and mutagenesis to probe the structure–function relationships of CloA. The results presented here revealed that *S. cerevisiae* can be used as viable hosts for functional expression and study of CloA orthologs. It was shown in this study that CloA orthologs from different fungi species exhibit differing oxidation profiles. Some orthologs in this study were observed to catalyze a one-step oxidation reaction, while others were able to catalyze a two-step or even a three-step oxidation. Several chimeric CloA variants were created in this study and were characterized using a combination of alanine scanning mutagenesis, loop-swapping, and loop-deletion experiments. These mutants revealed key residues in and around the F-G loop of the enzyme that is thought to play an important role in substrate recognition and potentially highlight the importance of membrane association. The chimeric CloAs were also shown to produce paspalic or lysergic acid at levels comparable or even better than wildtype CloA orthologs. These improved variants will be useful for incorporation into the biosynthetic pathway for the industrial production of ergot alkaloids.

## Data availability statement

The original contributions presented in the study are included in the article/Supplementary material, further inquiries can be directed to the corresponding author.

## Author contributions

LL and WY conceived, designed the study, and performed the experiments in this study. GW and MG performed the sequence similarity network analysis and ortholog selection. LL, GW, and MG analyzed the mass spectrometry data and wrote the manuscript. MG and WY revised the manuscript. All authors contributed to the article and approved the submitted version.

## Funding

This work was supported by grants from A*STAR under its Industry Alignment Fund-Industry Collaboration Project (IAF-ICP) Grant No I2001E0068 and the National Research Foundation (NRF) Synthetic Biology Research and Development Program Grant No. SBP-P3.

## Conflict of interest

The authors declare that the research was conducted in the absence of any commercial or financial relationships that could be construed as a potential conflict of interest.

## Publisher’s note

All claims expressed in this article are solely those of the authors and do not necessarily represent those of their affiliated organizations, or those of the publisher, the editors and the reviewers. Any product that may be evaluated in this article, or claim that may be made by its manufacturer, is not guaranteed or endorsed by the publisher.

## References

[ref1] Agurell S. Ramstad E. (1962). Biogenetic interrelationships of ergot alkaloids. Arch. Biochem. Biophys. 98, 457–470. doi: 10.1016/0003-9861(62)90212-6, PMID: 14036599

[ref2] Baylon J. L. Lenov I. L. Sligar S. G. Tajkhorshid E. (2013). Characterizing the membrane-bound state of cytochrome P450 3A4: structure, depth of insertion, and orientation. J. Am. Chem. Soc. 135, 8542–8551. doi: 10.1021/ja4003525, PMID: 23697766 PMC3682445

[ref3] Capozzi A. Scambia G. Pontecorvi A. Lello S. (2015). Hyperprolactinemia: pathophysiology and therapeutic approach. Gynecol. Endocrinol. 31, 506–510. doi: 10.3109/09513590.2015.1017810, PMID: 26291795

[ref4] Gerlt J. A. Bouvier J. T. Davidson D. B. Imker H. J. Sadkhin B. Slater D. R.. (2015). Enzyme function initiative-enzyme similarity tool (EFI-EST): a web tool for generating protein sequence similarity networks. Biochim. Biophys. Acta 1854, 1019–1037. doi: 10.1016/j.bbapap.2015.04.015, PMID: 25900361 PMC4457552

[ref5] Gietz R. D. Schiestl R. H. (2007). Large-scale high-efficiency yeast transformation using the LiAc/SS carrier DNA/PEG method. Nat. Protoc. 2, 38–41. doi: 10.1038/nprot.2007.15, PMID: 17401336

[ref6] Haarmann T. Machado C. Lübbe Y. Correia T. Schardl C. L. Panaccione D. G.. (2005). The ergot alkaloid gene cluster in Claviceps purpurea: extension of the cluster sequence and intra species evolution. Phytochemistry 66, 1312–1320. doi: 10.1016/j.phytochem.2005.04.011, PMID: 15904941

[ref7] Haarmann T. Ortel I. Tudzynski P. Keller U. (2006). Identification of the cytochrome P450 monooxygenase that bridges the clavine and ergoline alkaloid pathways. Chembiochem 7, 645–652. doi: 10.1002/cbic.200500487, PMID: 16538694

[ref8] Hsu J. C. Anderson J. A. (1970). Agroclavine hydroxylase of Claviceps purpurea. J. Chem. Soc. 20:1318. doi: 10.1039/c29700001318 4397222

[ref9] Kelley L. A. Mezulis S. Yates C. M. Wass M. N. Sternberg M. J. E. (2015). The Phyre2 web portal for protein modeling, prediction and analysis. Nat. Protoc. 10, 845–858. doi: 10.1038/nprot.2015.053, PMID: 25950237 PMC5298202

[ref10] Kim I.-S. Kim S.-U. Anderson J. A. (1981). Microsomal agroclavine hydroxylase of Claviceps species. Phytochemistry 20, 2311–2314. doi: 10.1016/S0031-9422(00)82653-9

[ref11] Liu H. Jia Y. (2017). Ergot alkaloids: synthetic approaches to lysergic acid and clavine alkaloids. Nat. Prod. Rep. 34, 411–432. doi: 10.1039/C6NP00110F, PMID: 28300233

[ref12] Michener J. K. Nielsen J. Smolke C. D. (2012). Identification and treatment of heme depletion attributed to overexpression of a lineage of evolved P450 monooxygenases. Proc. Natl. Acad. Sci. 109, 19504–19509. doi: 10.1073/pnas.1212287109, PMID: 23129650 PMC3511110

[ref13] Robinson S. L. Panaccione D. G. (2014). Heterologous expression of lysergic acid and novel ergot alkaloids in aspergillus fumigatus. Appl. Environ. Microbiol. 80, 6465–6472. doi: 10.1128/AEM.02137-14, PMID: 25107976 PMC4178656

[ref14] Schiff P. L. (2006). Ergot and its alkaloids. Am. J. Pharm. Educ. 70:98. doi: 10.5688/aj700598, PMID: 17149427 PMC1637017

[ref15] Shannon P. Markiel A. Ozier O. Baliga N. S. Wang J. T. Ramage D.. (2003). Cytoscape: a software environment for integrated models of biomolecular interaction networks. Genome Res. 13, 2498–2504. doi: 10.1101/gr.1239303, PMID: 14597658 PMC403769

[ref16] Šrejber M. Navrátilová V. Paloncýová M. Bazgier V. Berka K. Anzenbacher P.. (2018). Membrane-attached mammalian cytochromes P450: an overview of the membrane's effects on structure, drug binding, and interactions with redox partners. J. Inorg. Biochem. 183, 117–136. doi: 10.1016/j.jinorgbio.2018.03.002, PMID: 29653695

[ref17] Winblad B. Fioravanti M. Dolezal T. Logina I. Milanov I. G. Popescu D. C.. (2008). Therapeutic use of Nicergoline. Clin. Drug Investig. 28, 533–552. doi: 10.2165/00044011-200828090-00001 18666801

[ref18] Yang W. Bell S. G. Wang H. Zhou W. Bartlam M. Wong L.-L.. (2010). The structure of CYP101D2 unveils a potential path for substrate entry into the active site. Biochem. J. 433, 85–93. doi: 10.1042/BJ20101017 20950270

