# Supplementary material for: Increasing lysergic acid levels for ergot alkaloid biosynthesis: Directing catalysis via the F-G loop of Clavine oxidases
Source: Front Microbiol. 2023 Mar 16;14:1150937. doi: 10.3389/fmicb.2023.1150937 (PMC10060963; doi:10.3389/fmicb.2023.1150937)
Supplement: Supplementary file 1 [file Data_Sheet_1.DOCX]

Supplementary Material

**Supplementary tables and figures**

Table S1: Primers used for cloning of AT5 cloA mutants and loop alanine scanning mutagenesis

| No. | Primer name | Primer Sequence (5' → 3') |
| --- | --- | --- |
| 1 | AT5InsF | GCTTGGTACCGAGCTCGGATCCAAAAAAATGGAAAACAACTTCACTACAG |
| 2 | AT5InsR | CTTACCTTCGAAGGGCCCTCTAGACTCGAGATTCCATCTATCTCTCAACAATTTAACTTC |
| 3 | AT59HypoInsF | GTTACTTACCCAATGGTTCCAATGTTGTTAGGTTTAATTCCAGGTACATCTTTTGCTAAG |
| 4 | AT59HypoInsR | CCTAACAACATTGGAACCATTGGGTAAGTAACGAACAACCATGATTGAGTCATCAATTG |
| 5 | AT5CpurInsF | CCAATGGTTCCAATGGTTTTGTCATTAATTCCAGGTACATCTTTTGCTAAGTTTAATTC |
| 6 | AT5CpurInsR | GACAAAACCATTGGAACCATTGGATATGTAACGAACAACCATGATTGAGTCATCAATTG |
| 7 | AT5CpasInsF | CCACAATTGTTAGCTGTTTTGTCTTTAATTCCAGGTACATCTTTTGCTAAGTTTAATTC |
| 8 | AT5CpasInsR | GACAAAACAGCTAACAATTGTGGATAACCTAAAAACAACCATGATTGAGTCATCAATTG |
| 9 | AT5NloInsF | CCAATGATGGGTATGATCTTGGGTTTGATTCCAGGTACATCTTTTGCTAAGTTTAATTC |
| 10 | AT5NloInsR | CCCAAGATCATACCCATCATTGGGTAGTTGATCATCAACCATGATTGAGTCATCAATTG |

**Table S2: Description of cloA orthologues used in this study**

| **Number** | **UniprotID** | **Author's Identifier** | **SSN Cluster** | **Organism** | **Uniprot Status** | **Description** |
| --- | --- | --- | --- | --- | --- | --- |
| **1** | M1WEN7 | Cpur | Elymoclavine Monoxygenase | *Claviceps purpurea (strain 20.1)* | Reviewed - Evidence at the protein level | Cytochrome P450 monooxygenase cloA |
| **2** | G8GV80 | Cpas | Elymoclavine Monoxygenase | *Claviceps paspali* | Unreviewed - Protein inferred from homology | Elymoclavine monooxygenase |
| **3** | B9VR27 | Nlo | Elymoclavine Monoxygenase | *Epichloe festucae* | Unreviewed - Protein inferred from homology | Elymoclavine monooxygenase |
| **4** | E9EAT5 | AT5 | Elymoclavine Monoxygenase | *Metarhizium acridum (strain CQMa 102)* | Unreviewed - Protein inferred from homology | Putative elymoclavine monooxygenase |
| **5** | A8C7R4 | C7R4 | Elymoclavine Monoxygenase | *Claviceps fusiformis* | Reviewed - Protein inferred from homology | Inactive cytochrome P450 monooxygenase cloA |
| **6** | R9VXN6 | XN6 | Elymoclavine Monoxygenase | *Epichloe coenophiala* | Unreviewed - Protein inferred from homology | Elymoclavine monooxygenase |
| **7** | R9W253 | 253 | Elymoclavine Monoxygenase | *Epichloe coenophiala* | Unreviewed - Protein inferred from homology | Elymoclavine monooxygenase |
| **8** | G9FM49 | 9Hypo | Elymoclavine Monoxygenase | *Periglandula ipomoeae* | Unreviewed - Protein inferred from homology | Elymoclavine monooxygenase |
| **12** | E9F392 | 392 | Elymoclavine Monoxygenase | *Metarhizium robertsii (strain ARSEF 23 / ATCC MYA-3075)* | Unreviewed - Protein inferred from homology | Cytochrome P450 CYP5335A1 |
| **9** | M7UEF9 | BOTF1 | Putative elymoclavine monooxygenase | *Botryotinia fuckeliana (strain BcDW1)* | Unreviewed - Protein inferred from homology | Putative elymoclavine monooxygenase protein |
| **10** | T0KET3 | ET3 | Mixed Monoxygenase | *Colletotrichum gloeosporioides (strain Cg-14)* | Unreviewed - Protein inferred from homology | Cytochrome P450 |
| **11** | T0KX90 | X90 | Mixed Monoxygenase | *Colletotrichum gloeosporioides (strain Cg-14)* | Unreviewed - Protein inferred from homology | Cytochrome P450 |
| **13** | M1W6N5 | W6N5 | Benzoate 4-monooxygenase | *Claviceps purpurea (strain 20.1)* | Unreviewed - Protein inferred from homology | Related to benzoate 4-monooxygenase cytochrome P450 |
| **14** | M1VZB1 | ZB1 | Benzoate 4-monooxygenase | *Claviceps purpurea (strain 20.1)* | Unreviewed - Protein inferred from homology | Related to benzoate 4-monooxygenase cytochrome P450 |
| **15** | E9DSJ7 | SJ7 | Benzoate 4-monooxygenase | *Metarhizium acridum* | Unreviewed - Protein inferred from homology | Cytochrome P450, putative |

**Table S3: Top ten Phyre2 generated models of AT5 9Hypo CloA.** Columns of the results table shows the PDB ID of the templates of known structure, the confidence level, sequence identity and template information.

| No. | Template (PDB ID) | Confidence score (%) | Percent identity | Template information |
| --- | --- | --- | --- | --- |
| 1 | 4LXJ | 100 | 14 | PDB molecule: Lanosterol 14-alpha demethylase PDB title: *Saccharomyces cerevisiae* lanosterol 14-alpha demethylase with 2 lanosterol bound |
| 2 | 3NA0 | 100 | 18 | PDB molecule: cholesterol side-chain cleavage enzyme, mitochondrial PDB title: Crystal structure of human CYP11a1 in complex with 20.22-2 dihydroxycholesterol |
| 3 | 5T6Q | 100 | 15 | PDB molecule: Cytochrome P450 4B1 PDB title: Structure of cytochrome P450 4B1 (4B1) complexed with octane: an n-2 alkane and fatty acid omega-hydroxylase with a covalently bound heme |
| 4 | 1TQN | 100 | 19 | PDB molecule: Cytochrome P450 PDB title: Cytochrome P450 |
| 5 | 4FDH | 100 | 19 | PDB molecule: Cytochrome P450 11B2, mitochondrial PDB title: structure of human aldosterone synthase, CYP11B2, in complex with 2 fadrozole |
| 6 | 2X2N | 100 | 16 | PDB molecule: Lanosterol 14 alpha-demethylase PDB title: X-Ray structure of CYP51 from *Trypanosoma brucei* in complex with posaconazole in two different conformations. |
| 7 | 3K9V | 100 | 18 | PDB molecule: 1, 25-dihydroxyvitamin d(3) 24-hydroxylase, mitochrondrial PDB title: Crystal structure of rat mitochondrial P450 24A1 S57D in complex with 2 chaps |
| 8 | 3DAN | 100 | 15 | PDB molecule: Cytochrome P450 74A2 PDB title: Crystal structure of allene oxide synthase |
| 9 | 3CZH | 100 | 16 | PDB molecule: Cytochrome P450 PDB title: Cytochrome P450 |
| 10 | 6FYJ | 100 | 12 | PDB molecule: Fatty-acid peroxygenase PDB title: Cytochrome P450 peroxygenase CYP152K6 in complex with myristic acid |

**Table S4. Summary of the activity of wildtype CloA and loop deletion mutants in the presence of agroclavine**

|  | Mean Concentration of Agroclavine (µM∙OD_600_ ^-1^) (± ^α^SD) | Mean Concentration of Elymoclavine  (µM∙OD_600_ ^-1^) (± ^α^SD) | Mean Concentration of Lysergic acid (µM∙OD_600_ ^-1^) (± ^α^SD) |
| --- | --- | --- | --- |
| pYES2 | 4.12 (± 0.263) | N.D* | N.D* |
| Cpur | N.D* | 0.128 (± 0.024) | 2.23 (± 0.350) |
| Cpur DeLoop | 2.83 (± 0.22) | N.D* | N.D* |
| Cpas | N.D* | 1.73 (± 0.231) | 0.398 (± 0.069) |
| Cpas DeLoop | 3.27 (± 0.20) | N.D* | N.D* |
| Nlo | N.D* | 0.89 (± 0.219) | 1.03 (± 0.164) |
| Nlo DeLoop | 2.98 (± 0.22) | N.D* | N.D* |
| 9Hypo | N.D* | 2.23 (± 0.173) | 0.174 (± 0.014) |
| 9Hypo DeLoop | 3.04 (± 0.273) | N.D* | N.D* |
| 253 | 1.98 (± 0.158) | 0.798 (± 0.158) | N.D* |
| 253 DeLoop | 3.57 (± 0.469) | N.D* | N.D* |
| C7R4 | 0.118 (± 0.077) | 3.40 (± 0.588) | N.D* |
| C7R4 DeLoop | 2.73 (± 0.138) | N.D* | N.D* |
| XN6 | N.D* | 0.739 (± 0.145) | 1.13 (± 0.115) |
| XN6 DeLoop | 2.92 (± 0.213) | N.D* | N.D* |
| ^α^SD, denotes standard deviation of three biological replicates | | | |
| N.D*, denotes no detectable activity in the presence of agroclavine | | | |


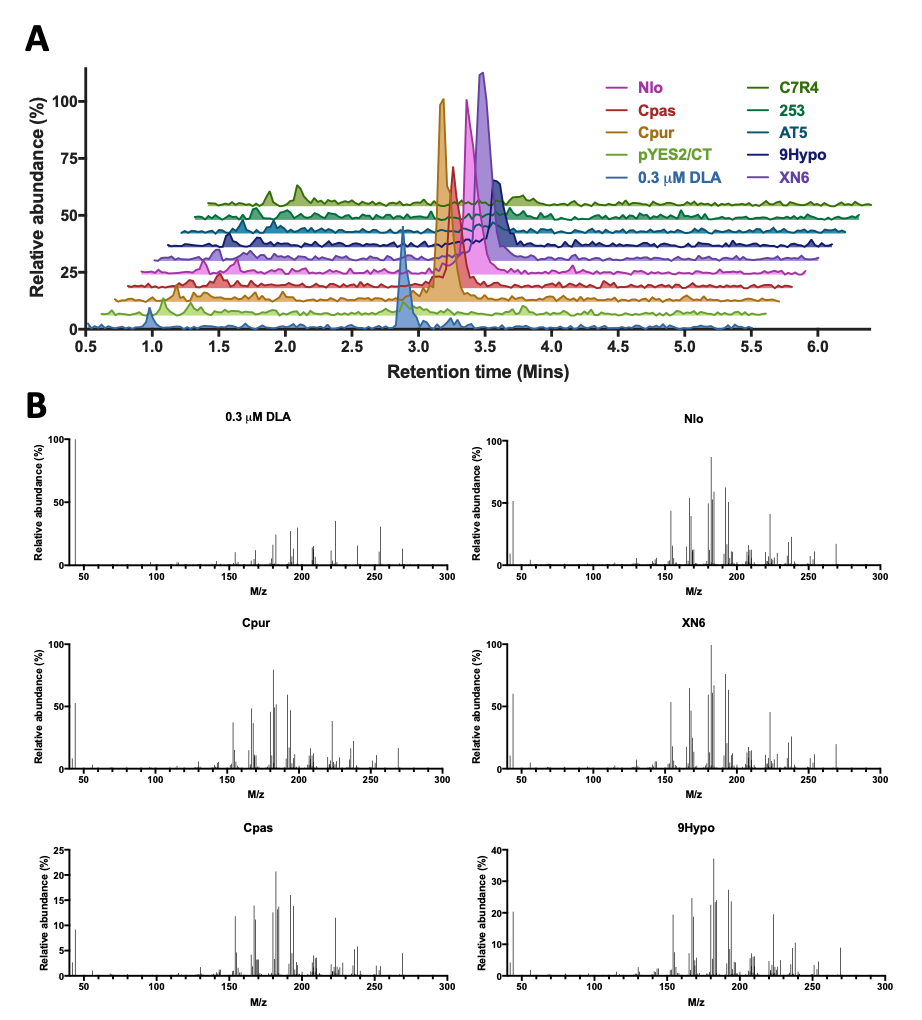


**Figure S1: LC-MS/MS analysis of wildtype CloA products in the presence of agroclavine**. (A) TIC of [M+H]^+^ mass of 269.1285 m/z corresponding to lysergic acid produced by wildtype CloA. (B) MS/MS fragmentation patterns from the samples that eluted peaks on the TIC of [M+H]^+^ = 269.1285, that correspond to the eluted peaks of the DLA standard,


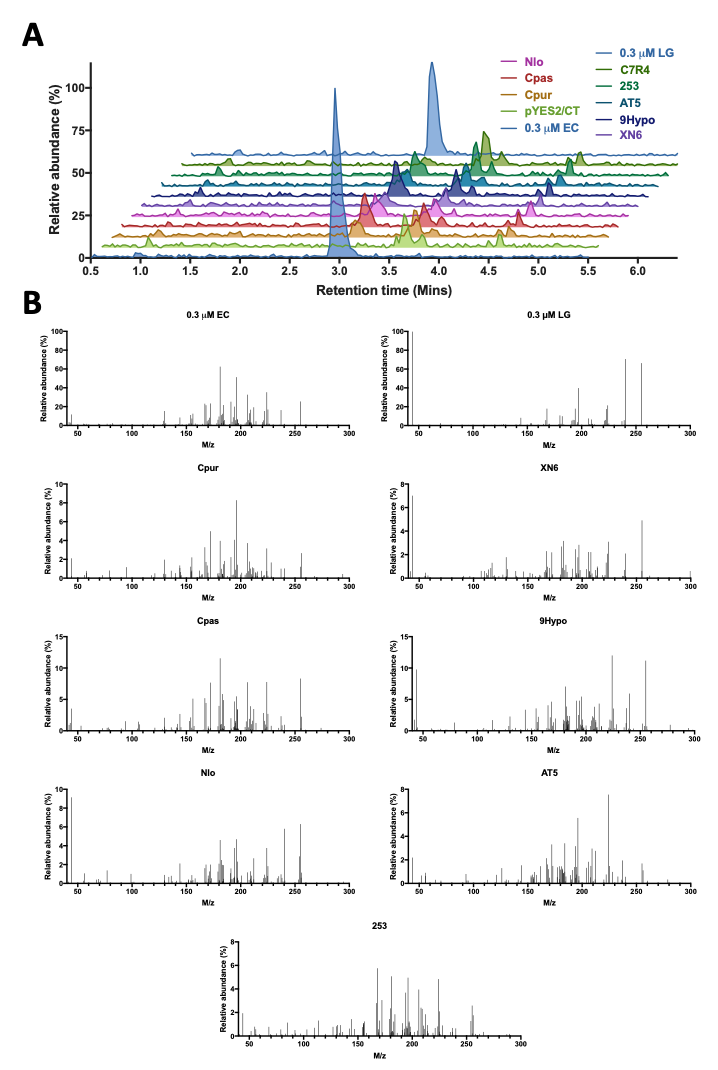


**Figure S2: LC-MS/MS analysis of wildtype CloA products in the presence of agroclavine**. (A) TIC of [M+H]^+^ mass of 255.1492 m/z corresponding to elymoclavine (EC) or lysergol (LG) produced by wildtype CloA. (B) MS/MS fragmentation patterns from the samples that eluted peaks on the TIC of [M+H]^+^ = 255.1492 m/z, that correspond to the eluted peaks of the EC and LG standard.

**
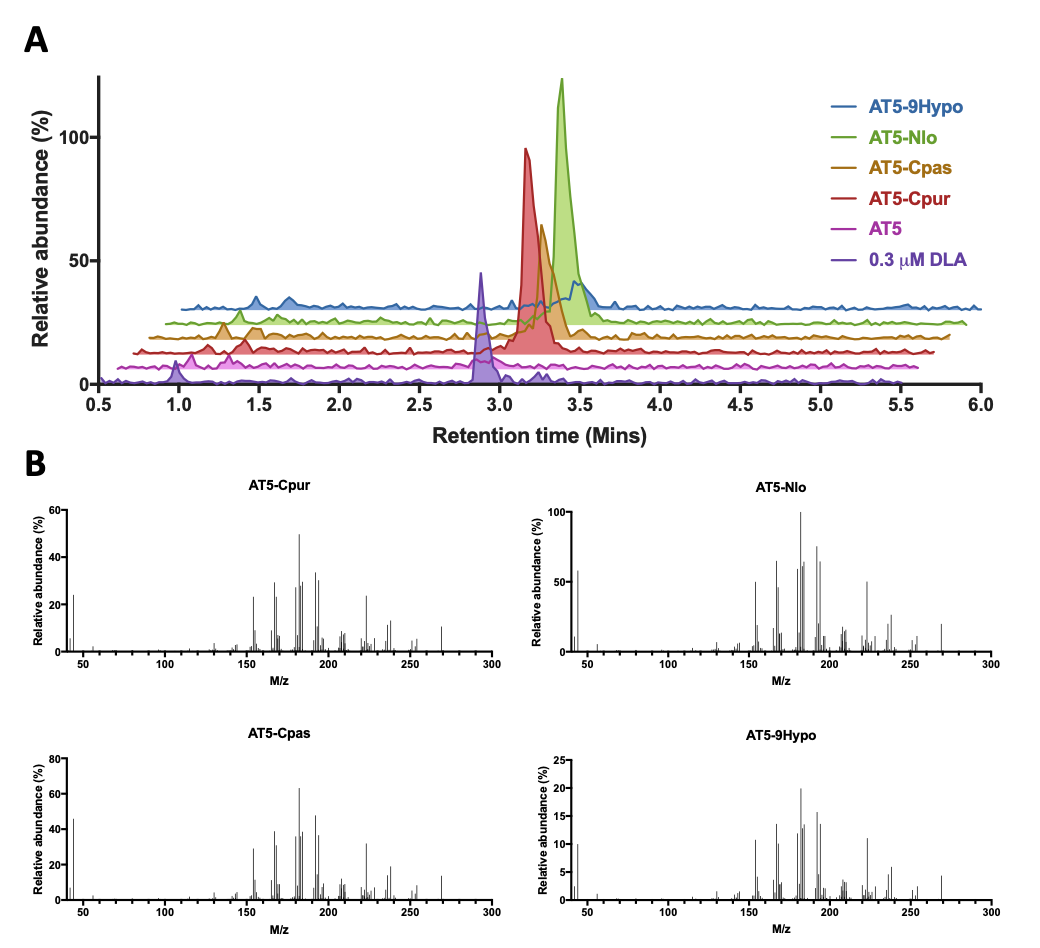
**

**Figure S3: LC-MS/MS analysis of products from AT5 CloA loop-swapping mutants in the presence of agroclavine**. (A) TIC of [M+H]^+^ mass of 255.1492 m/z corresponding to elymoclavine (EC) or lysergol (LG) produced by wildtype CloA. (B) MS/MS fragmentation patterns from the samples that eluted peaks on the TIC of [M+H]^+^ = 269.1285 m/z, that correspond to the eluted peaks of the DLA standard,


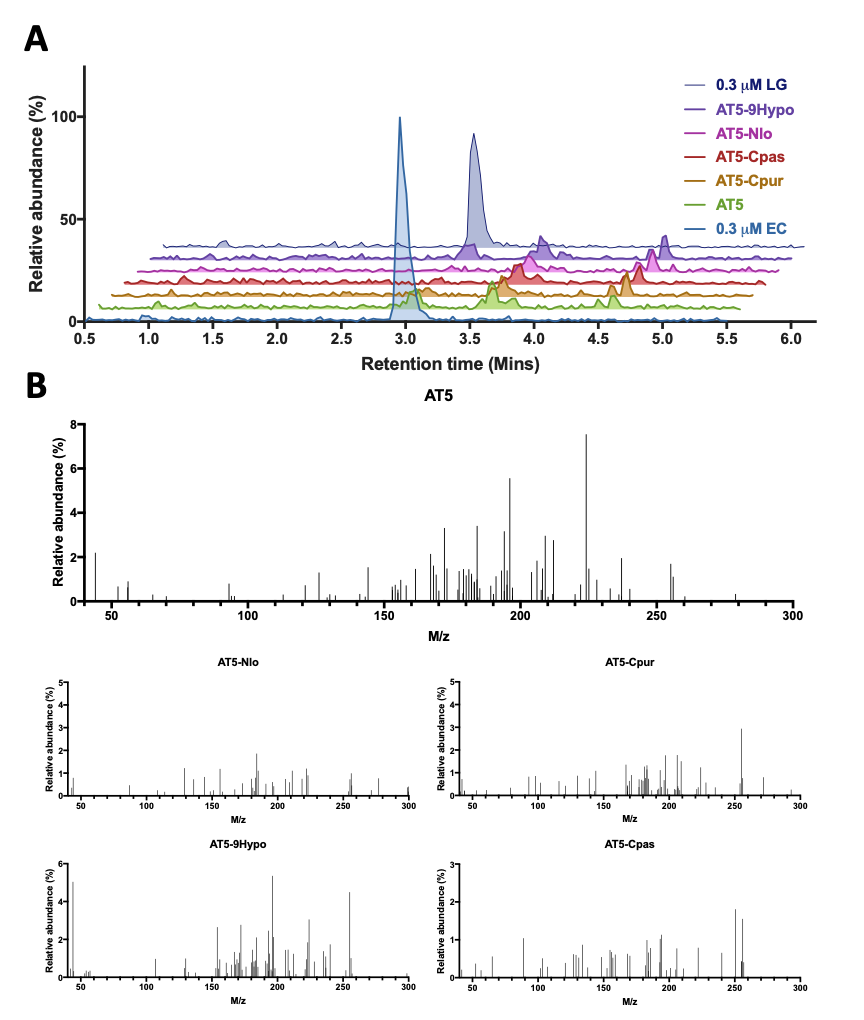


**Figure S4. LC-MS/MS analysis of products from AT5 CloA loop-swapping mutants in the presence of agroclavine**. (A) TIC of [M+H]^+^ mass of 255.1492 m/z corresponding to elymoclavine (EC) or lysergol (LG) produced by wildtype CloA. (B) MS/MS fragmentation patterns from the samples that eluted peaks on the TIC of [M+H]^+^ = 255.1492 m/z, that correspond to the eluted peaks of the EC and LG standard.


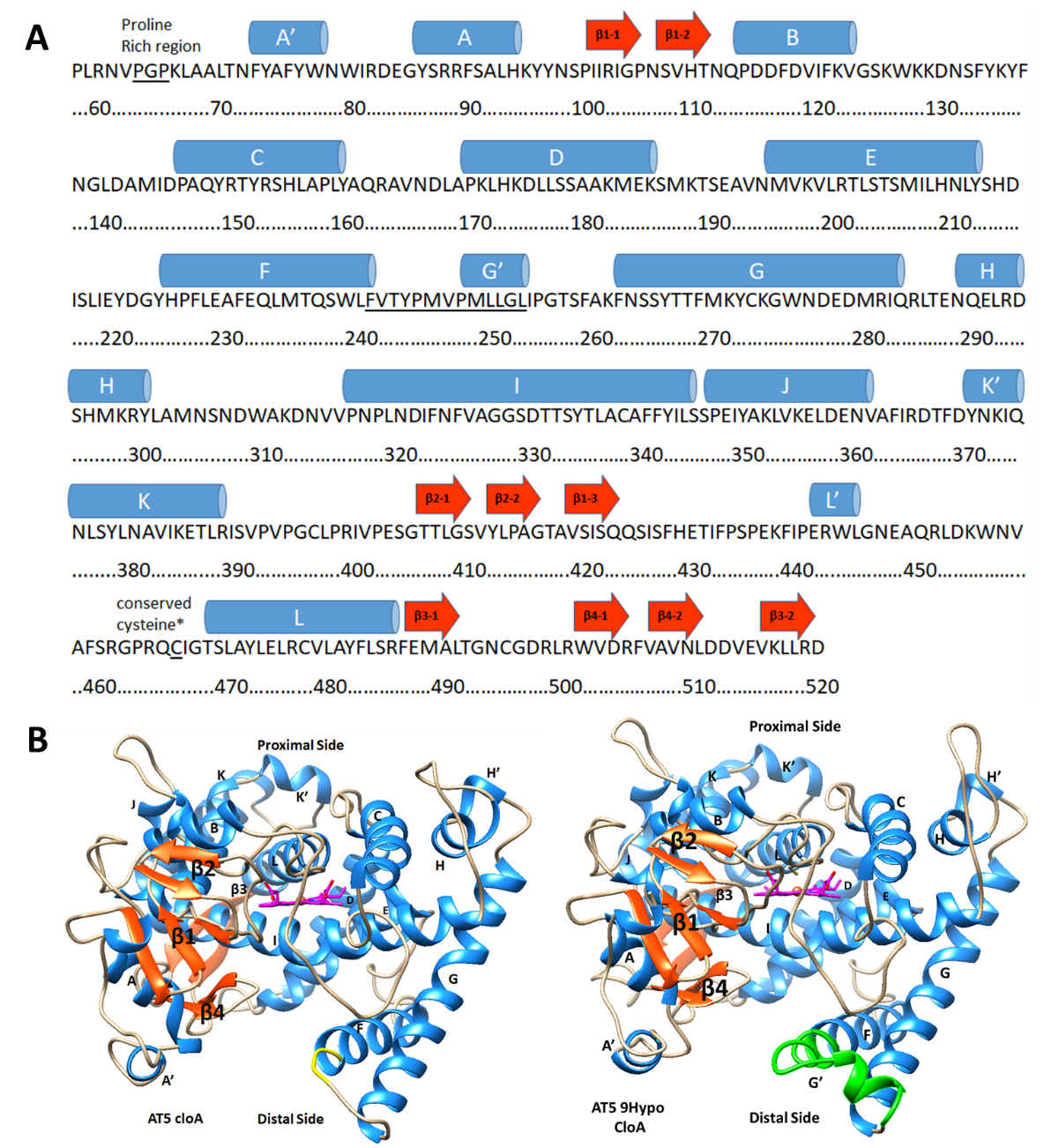


**Figure S5: Phyre2 generated structures of AT5 and AT5 9Hypo cloA**. (A) Secondary structure of the AT5 9Hypo cloA model predicted by Phyre2. Cylinders represents helices while arrows represent β-sheets. Minor helices are indicated by an apostrophe. The inserted sequence from 9Hypo cloA is underlined. (B) Ribbon representation of AT5 cloA (left) and AT5 9Hypo cloA (right). The heme prosthetic group is colored magenta, helices in blue and β-sheets in orange. The region between the conserved residues WL and IP are highlighted yellow (AT5 cloA) and in green (AT5 9Hypo cloA).


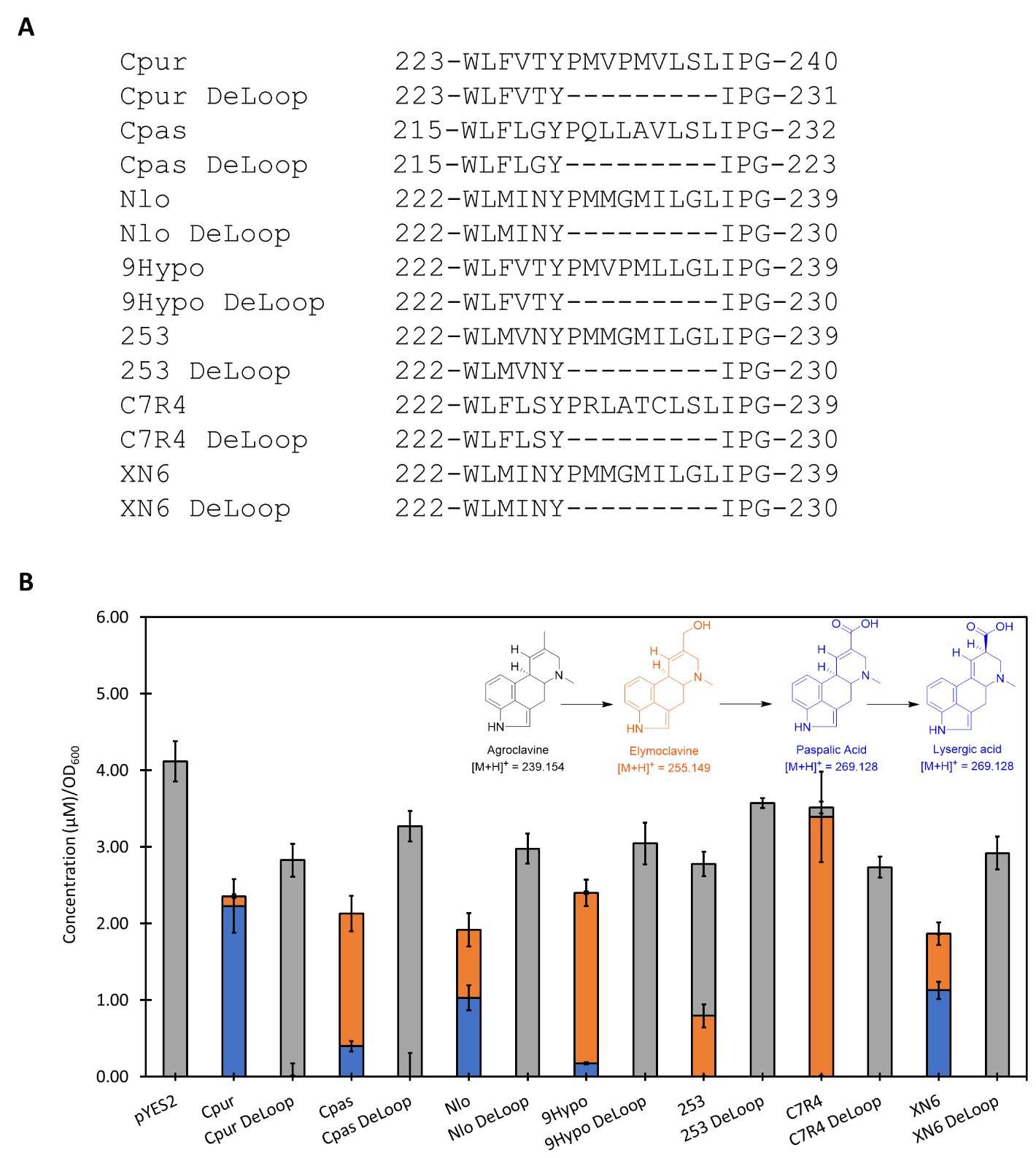


**Figure S6: Screening of wildtype CloA and loop deletion mutants in the presence of agroclavine.** (A) Sequence alignment of wildtype and DeLoop mutants. (B) Blue bars represent the mean concentration of paspalic or lysergic acid while orange bars represent the mean concentration of elymoclavine produced by AT5 9Hypo CloA phenylalanine gate mutants. Error bars are representative of three biological replicates.
